# Propofol versus Midazolam for Upper Gastrointestinal Endoscopy in Cirrhotic Patients: A Meta-Analysis of Randomized Controlled Trials

**DOI:** 10.1371/journal.pone.0117585

**Published:** 2015-02-03

**Authors:** Hsiao-Chien Tsai, Yu-Cih Lin, Ching-Lung Ko, Horng-Yuan Lou, Ta-Liang Chen, Ka-Wai Tam, Chien-Yu Chen

**Affiliations:** 1 Department of Anesthesiology, Taipei Medical University Hospital, Taipei Medical University, Taipei, Taiwan; 2 Graduate Institute of Nursing, College of Nursing, Taipei Medical University, Taipei, Taiwan; 3 Division of Gastroenterology and Hepatology, Department of Internal Medicine, Taipei Medical University, Taipei, Taiwan; 4 Department of Anesthesiology, School of Medicine, College of Medicine, Taipei Medical University, Taipei, Taiwan; 5 Division of General Surgery, Department of Surgery, Taipei Medical University—Shuang Ho Hospital, New Taipei City, Taiwan; 6 Department of Surgery, School of Medicine, College of Medicine, Taipei Medical University, Taipei, Taiwan; 7 Center for Evidence-based Medicine, Taipei Medical University, Taipei, Taiwan; 8 Center for Evidence-based Health Care, Taipei Medical University-Shuang Ho Hospital, New Taipei City, Taiwan; 9 Graduate Institute of Humanities in Medicine, Taipei Medical University, Taipei, Taiwan

## Abstract

**Background:**

Sedation during gastrointestinal endoscopy is often achieved using propofol or midazolam in general population. However, impaired protein synthesis, altered drug metabolism, and compromised hepatic blood flow in patients with liver cirrhosis might affect the pharmacokinetics of sedatives, placing cirrhotic patients undergoing endoscopy at a greater risk of adverse events. The objective of this study was to assess comparative efficacies and safety of propofol and midazolam in cirrhotic patients undergoing endoscopy.

**Methods:**

Randomized, controlled trials comparing propofol with midazolam in cirrhotic patients undergoing gastrointestinal endoscopy were selected. We performed the meta-analysis, using a random-effect model, the Review Manager, Version 5.2, statistical software package (Cochrane Collaboration, Oxford, UK) according to the PRISMA guidelines.

**Results:**

Five studies between 2003 and 2012, including 433 patients, were included. Propofol provided a shorter time to sedation (weight mean difference: -2.76 min, 95% confidence interval: -3.00 to -2.51) and a shorter recovery time (weight mean difference -6.17 min, 95% confidence interval: -6.81 to -5.54) than midazolam did. No intergroup difference in the incidence of hypotension, bradycardia, or hypoxemia was observed. Midazolam was associated with the deterioration of psychometric scores for a longer period than propofol.

**Conclusion:**

This meta-analysis suggests that Propofol sedation for endoscopy provides more rapid sedation and recovery than midazolam does. The risk of sedation-related side effects for propofol does not differ significantly from that of midazolam. The efficacy of propofol in cirrhotic patients undergoing endoscopy is superior to those of midazolam.

## Introduction

Patients with liver cirrhosis undergo upper gastrointestinal endoscopy (UGIE) repeatedly during screening for adverse events related to portal hypertension, such as esophageal varices. To reduce patient discomfort, UGIE is frequently performed under sedation. However, impaired hepatic function and hepatic encephalopathy in cirrhotic patients increase the challenge and risk of sedation. Enhanced sedative effects caused by a higher plasma level of the drug or prolonged sedative effects caused by delayed clearance might cause cardiopulmonary adverse events and acute deterioration of hepatic encephalopathy, which might delay recovery.[1]

The most common sedative regimens used for UGIE consist of midazolam or propofol alone with or without opioids [2,3]. Midazolam is superior to older benzodiazepines because of its rapid onset and potent amnestic properties. However, the half-life of midazolam is still prolonged in patients with liver failure [4–6]. The hypnotic agent propofol is initially redistributed to adipose tissue, reducing plasma concentration rapidly with short duration of action, then metabolized rapidly in the liver, and excreted by the kidney. Propofol is often used as an alternative to midazolam in patients with impaired hepatic or renal function because it requires no dose adjustment [7].

Because both midazolam and propofol are widely used sedate patients undergoing gastrointestinal endoscopy (GIE), numerous comparative trials have been performed to evaluate the efficacy and safety of these drugs [1,8–16]. Some of these trials failed to detect a statistically significant difference between midazolam and propofol because the samples were small. However, two systematic reviews and meta-analyses of randomized controlled trials (RCTs) indicated that the potential benefits of propofol sedation during GIE include a shorter recovery time, a shorter discharge time, higher postanesthesia recovery scores, greater sedation, and improved patient cooperation compared with midazolam sedation, with no increase in adverse events [17,18]. However, whether propofol provides the same benefits in high-risk groups, such as patients with liver cirrhosis, remains unclear. The primary aim of our systematic review was to compare the safety and efficacy of using propofol and midazolam to sedate cirrhotic patients undergoing UGIE.

## Methods

### Data Sources and Search Strategy

Relevant studies were identified by conducting a computer search of the PubMed, EMBASE, Scopus, and Cochrane databases. The following Medical Subject Headings search headings were used for the search: *sedation*, *anesthesia*, *propofol*, *midazolam* OR *dormicum* OR *hypnovel and versed* OR *benzodiazepine*, *liver cirrhosis* OR *hepatic failure* OR *hepatic encephalopathy*, *upper gastrointestinal endoscopy* OR *esophago-gastro-duodenoscopy* OR *panendoscopy* OR *gastroscopy* OR *diagnostic endoscopic procedure*. These terms and the combinations of these terms were also used in keyword searches. To broaden the search, the “related articles” search feature provided by PubMed was used. All of the retrieved abstracts, studies, citations were reviewed. We identified additional studies by manually searching the reference sections and contacting experts in the field. Unpublished studies were identified by searching the ClinicalTrials.gov registry (http://clinicaltrials.gov/). The final search was performed in November 2014 without language restrictions. The current systematic review was accepted by the international Prospective Register of Ongoing Systematic Reviews of the National Institutes of Health Research (PROSPERO CRD42014009652).

### Study Selection

Only RCTs that compared the use of propofol with the use of midazolam in sedating cirrhotic patients undergoing UGIE were included in our study. RCTs that (1) compared a propofol-based sedative regimen with a midazolam-based regimen, (2) examined patients with any stage of cirrhosis undergoing UGIE, and (3) addressed the incidence of sedation-related side effects or drug-related adverse events as outcomes of interest were included in the analysis. Studies meeting at least one of the following criteria were excluded from our analysis: (1) the patients were younger than 18 years of age; (2) other types of endoscopic examinations, such as colonoscopy, were evaluated; (3) or the appropriate data could not be extracted or calculated based on the published results. When duplicate studies using the same data sets were published, the one with the larger population would be included.

### Data Extraction and Quality Assessment

Two review authors (KWT and CYC) extracted the following information independently: authors; year of publication; study design and population characteristics; inclusion and exclusion criteria; the anesthetic regimen used; the anesthetic performance; and any resulting adverse events, including hypoxemia, hypotension, and bradycardia. The two reviewers assessed the eligibility of the retrieved studies according to the inclusion criteria specified. All the data extracted then cross-checked to rule out the discrepancy. Any disagreements were discussed with a third reviewer (HCT), and resolved by consnesus. The authors of the retrieved studies were contacted for additional information if necessary.

### Methodological Quality Appraisal

The risk-of-bias method recommended by The Cochrane Collaboration was used to assess the quality of the included RCTs[19]. The following domains were assessed: allocation concealment; allocation generation; blinding adequacy; completeness of outcome data; freedom from selective reporting and other biases.

### Data Synthesis and Statistical Analysis

We used the following outcomes to evaluate the anesthetic performance of propofol and midazolam in cirrhotic patients during and after UGIE: (1) time to procedure; (2) time to recovery; (3) time to discharge; (4) procedure time; and (5) sedation-related adverse events, including hypotension, bradycardia, hypoxemia, and deterioration of encephalopathy. We conducted the meta-analysis using the Review Manager, Version 5.2, statistical software package (Cochrane Collaboration, Oxford, UK). The meta-analysis was performed according to the PRISMA guidelines [20,21]. When necessary, standard deviations were estimated using the confidence interval (CI) limits, the standard error, or the range values.

Risk ratios (RRs) were used as the summary statistic for the analysis of the dichotomous outcomes. The weighted mean difference (WMD) was determined for the continuous outcomes. The 95% CIs were determined for both the RRs and the WMDs. Pooled estimates of the RR and WMD were determined using the DerSimonian and Laird random-effect model [22], which provides an estimate of the average treatment effect in statistically heterogeneous studies and a large CI for conservative statistical claims.

To evaluate the statistical heterogeneity and the inconsistency of the treatment effects across the studies, the Cochrane *Q* test and the *I* ^*2*^ statistic were used, respectively. Statistical significance was set at 0.10 for the Cochrane *Q* tests. The proportion of the total outcome variability that was attributable to the variability across the studies was quantified as *I* ^*2*^. Sensitivity analyses were performed to assess any impact of study quality on the effect estimates.

## Results

### Trial Characteristics

**Fig 1:**
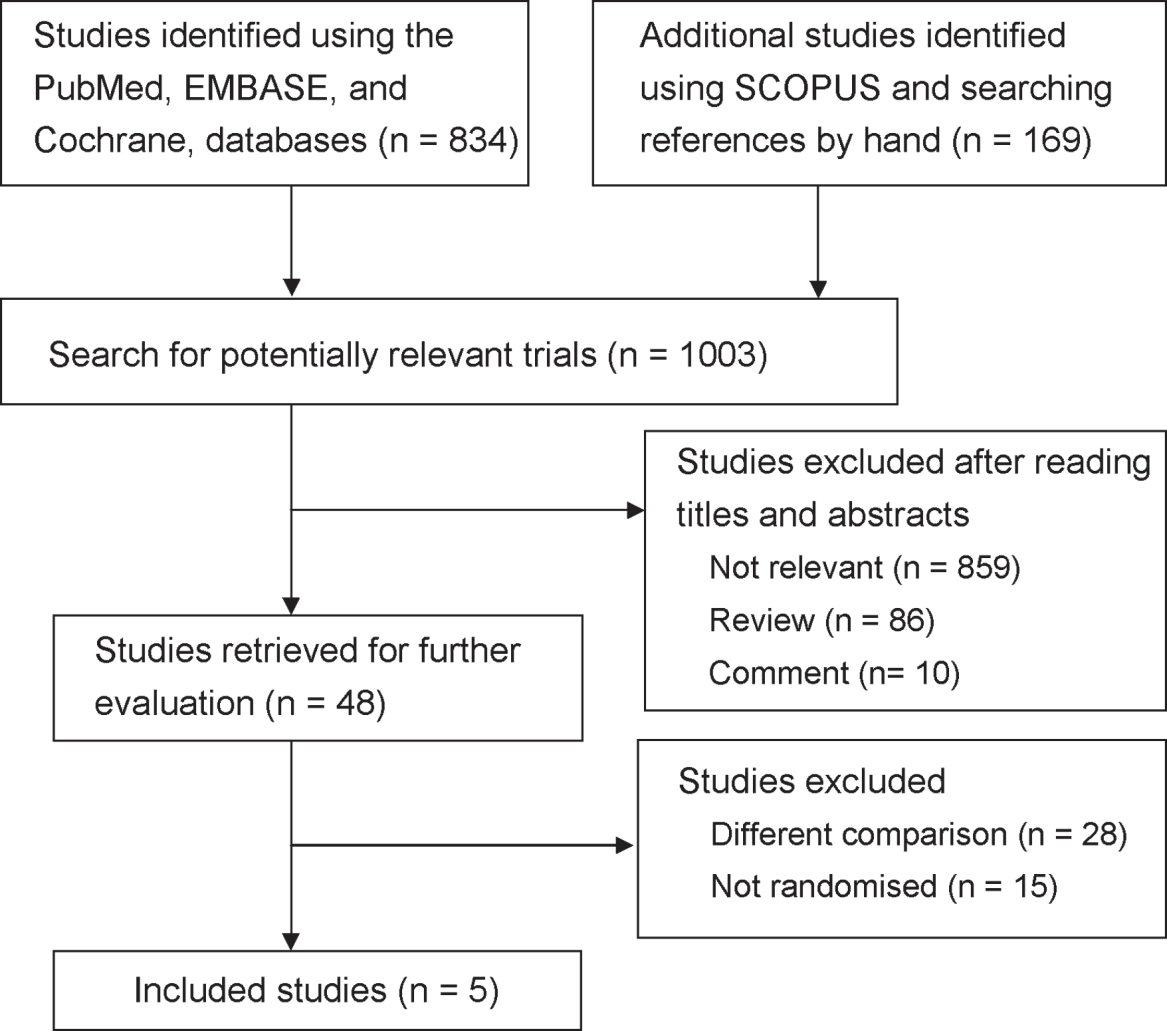
Flowchart of the study selection process.

Five RCTs that examined a total of 433 patients were selected for our meta-analysis. Fig. 1 shows the selection process used in our study. The initial search strategy yielded 1003 citations. Based on the screening criteria for titles and abstracts, 955 studies were excluded. After reviewing the full text of the remaining 48 reports, five eligible RCTs were subjected to further analysis [1,23–26].

These five RCTS were published in English between 2003 and 2012, and had sample sizes ranging from 20 to 210 patients. All of the five trials examined cirrhotic patients with American Society of Anesthesiologists (ASA) status II–III who underwent sedation with propofol or midazolam for UGIE. A propofol group was compared with a midazolam group in all of the trials. One RCT investigated the outcomes in cirrhotic patients who underwent UGIE without undergoing sedation [23]. In addition, one RCT compared the outcomes in a nonsedated group without cirrhosis [24], and one study compared the outcomes in nonsedated and noncirrhotic patients who were matched to the cirrhotic group [1]. Most of the patients included in the five selected RCTs were Child-Pugh (CP) classification A or B. Similar regimens were used, except the Weston study[25], which meperidine was used as rescue only in the midazolam group. Other variation among the studies was minimal,. The patient characteristics, anesthetic interventions, and inclusion and exclusion criteria are reported in Table 1.

**Table 1:** Characteristics of the selected randomized controlled trials.

Study	Inclusion and exclusion criteria	Patients (male:female)	Age, (y)^a^	CP score ^a^ ^,^ ^b^	Intervention
Agrawal	*Inclusion*: age, 18–70 y/o; liver cirrhosis	P: 40 (28:12)	P: 41.1 ± 9.6	P: 8.3 ± 1.6	P: 0.5–1 mg/kg and 10–20 mg as necessary
2012[23]	*Exclusion*: ASA IV-V, HCC, active GI bleeding, alcohol use, psychoactive drugs use, any neurologic diseases	M: 42 (31:11)	M: 40.1 ± 10.5	M: 8.5 ± 1.2	M: 0.5–1 mg at intervals of 1–3min as necessary (total 3–6 mg)
		Co: 45 (31:14)	Co: 41.9 ± 7.9	Co: 8.1 ± 1.8	Co: no sedation
Khamaysi 2011 [1]	*Inclusion*: age, 18~70 y/o; CP score 5–7 (CP class A-B)	P: 31 (14:17)	P: 57.3 ± 11.8	P: 6.6 ± 1.47	P: 30–50 mg and 10–20 mg as necessary (total 70–100 mg)
	*Exclusion*: active bleeding, alcohol or drug abuse, active neurological impairment	M: 30 (21:9)	M: 55.2 ± 12.6	M: 6.9 ± 1.56	M: 0.5–1mg at intervals of 1–3min as necessary (total 3–6 mg)
		Co: 30 (15:15)	Co: 56.1 ± 10.0	Co: 5.0 ±0.0	Co: noncirrhotic patients
Correia 2011 [26]	*Inclusion*: age, 18–75 y/o; liver cirrhosis (CP class A-C)	P: 100 (64:36)	P:54.12 ± 10.51	P: A:B:C = 70:22:8	P: 0.25 mg/kg and 20–30 mg as necessary (max: 400 mg) + fentanyl 50 μg
	*Exclusion*: emergency procedures, ASA IV-V, alcohol use, psychoactive drugs use, hepatic encephalopathy	M: 110 (84:26)	M:52.57 ±11.51	M: A:B:C = 82:23:5	M: 0.05 mg/kg and 1mg as necessary (max: 0.1 mg/kg or 10 mg) + fentanyl 50 μg
Riphaus 2009 [24]	*Inclusion*: age > 18 y/o; liver cirrhosis, inpatient	P: 40 (23:17)	P: 62.6 ± 11.4	P: A:B:C = 25:11:4	P: 40mg(<70 kg) or 60mg(>70 kg) and 10–20 mg as necessary
	*Exclusion*: ASA IV-V, active GI bleeding, alcohol use, psychoactive medication, hepatic encephalopathy	M: 20 (11:9)	M:61.5 ± 10.3	M: A:B:C = 13:5:2	M: 2.5 mg, repeated as necessary (maximum 7.5 mg)
		Co: 20 (12:8)	Co: 60.2 ± 10.8	Co: A:B:C = 0:0:0	Co: noncirrhotic patients, no sedation
Weston	*Inclusion*: ASA III, CP class A-B	P: 10 (7:3)	P: 53.9 ± 9.1	P: A:B:C = 6:4:0	P: 30~50 mg and 10–20 mg as necessary
2003 [25]	*Exclusion*: inpatient, active neurological impairment, alcohol/ drug abuse	M: 10 (5:5)	M:53.0 ± 8.2	M A:B:C = 5:5:0	M: incremental midazolam 0.5–1 mg and meperidine 12.5–25 mg as necessary

CP: Child-Pugh; Co: control; P: propofol; M: midazolam; HCC: hepatocellular carcinoma

^a^Data reported as the mean ± standard deviation

^b^Cirrhosis staging reported as A: < 7; B: 7–9; and C: > 9

Table 2 shows the results of the methodological quality assessment of the five selected RCTs. Three of them used acceptable methods of randomization [23,25,26], and one of them clearly described the method of allocation concealment [23]. All of them reported the blinding of the patients and the outcome assessors. All of the RCTs [1,23,25,26], except for the study by Riphaus et al. [24], based their analyses on the intention-to-treat principle. The number of patients lost to follow up was acceptable in all of the studies. None of the studies reported information that was relevant to determining incomplete data bias. Other biases included a significant difference in the incidence of esophageal varices and portal gastropathy between two of the groups in one study [1].

**Table 2:** Methodological quality assessment of selected trials.

Study	Country	Allocation generation	Allocation concealment	Blinding	Loss of follow-up, %	Data analysis	Other biases
Agrawal 2012 [23]	India	Computer-generated	Adequate	Assessor & patient blinded	0	ITT	Unclear
Khamaysi 2011 [1]	Israel	Unclear	Unclear	Assessor & patient blinded	0	ITT	P: more esophageal varices; M: more portal gastropathy
Correia 2011 [26]	Brazil	Random sequence	Unclear	Assessor & patient blinded	0	ITT	Unclear
Riphaus 2009 [24]	Germany	Unclear	Unclear	Assessor & patient blinded	8.33	PP	Unclear
Weston 2003 [25]	USA	Sealed envelope	Unclear	Assessor & patient blinded	0	ITT	Insufficient size

ITT: Intention-to-treat; PP: Per-protocol; P: propofol; M: midazolam

### Anesthetic Performance

**Fig 2:**
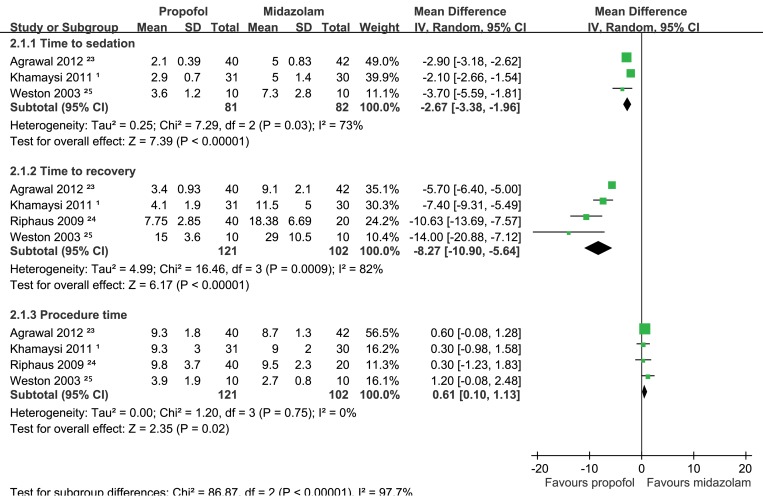
Forest plots of meta-analyses comparing the anesthetic performance of propofol to that of midazolam in RCTs of cirrhotic patients undergoing UGIE. Based on (1.1.1) time to sedation (1.1.2) time to recovery, or (1.1.3) procedure time.

**(i) Time to sedation.** Three of the selected RCTs [1,23,25] compared the time to sedation between propofol and midazolam based on the time required to achieve adequate sedation after first administration of the sedative and reported significantly shorter induction times in the propofol groups. The meta-analysis revealed that the time to sedation for propofol was shorter than that of midazolam (WMD: -2.67 min; 95% CI: -3.38 to-1.96; *p* < 0.00001; Fig. 2). Significant heterogeneity existed across these studies (*I* ^*2*^ = 73%, *p* = 0.03).

**(ii) Time to recovery.** One of the selected RCTs measured recovery based on a postanesthesia recovery score of 10 [17]. Three of the selected RCTs [1,23,25] evaluated time to recovery based on the following parameters: blood pressure and heart rate falling within 20% of baseline; oxygen saturation > 90% according to pulse oximetry, toleration of oral fluids, and ability to stand without assistance or per baseline function. One of these three studies [25] required full recovery of alertness based on the Observer’s Alertness and Assessment Scale [27]. The meta-analysis indicated that propofol provided a significantly shorter time to recovery than midazolam did (WMD: -8.27 min; 95% CI: -10.90 to-5.64; *p* < 0.00001; Fig. 2). In addition, we observed a high level of heterogeneity across the studies (*I* ^*2*^ = 82%; *p* = 0.0009).

.

**(iii) Procedure time.** Four of the selected RCTs [1,23–25] evaluated the period between endoscope insertion and withdrawal. No significant difference was observed between the propofol and midazolam groups in any of these studies. However, the meta-analysis revealed that the procedure time was significantly shorter when midazolam was used than when propofol was used (WMD: 0.61 min; 95% CI: 0.10 to 1.13; *p* = 0.02; Fig. 2).

**Fig 3:**
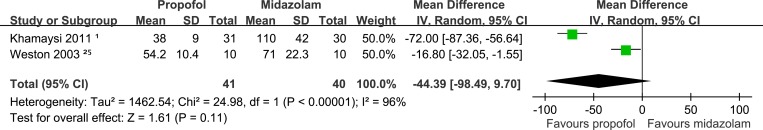
Forest plots of meta-analyses comparing the anesthetic performance of propofol to that of midazolam in RCTs of cirrhotic patients undergoing UGIE. Based on time to discharge.

**(iv) Time to discharge.** Two of the selected RCTs evaluated the time to discharge [1,25]. In these studies, the patient was discharged at the discretion of the recovery room nurse after the endoscopist had discussed procedure results with the patient and his or her family member. The time to discharge with propofol was significantly shorter than that of midazolam in one these studies (38.0 ± 9.0 vs 110.0 ± 42.0; *p* < 0.001) [1], whereas no significant difference was observed in the other study (54.2 ± 10.4 vs 71.0 ± 22.3; *p* = 0.07) [25]. The meta-analysis revealed no significant difference in time to discharge between the propofol and midazolam groups (WMD: -44.39 min; 95% CI: -98.49 to 9.7; *p* = 0.11; Fig. 3). A high level of heterogeneity across the studies was noted (*I* ^*2*^ = 96%; *p* < 0.00001).

### Adverse events

**Fig 4:**
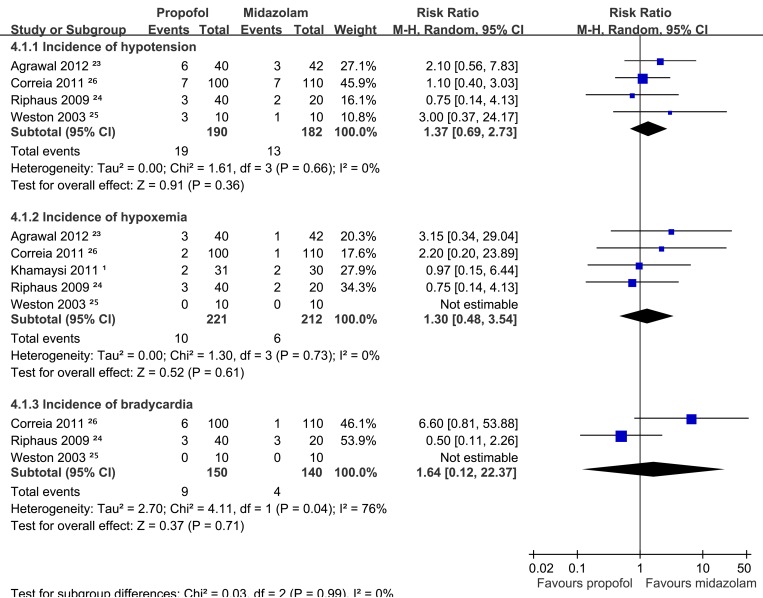
Forest plots of meta-analyses comparing the sedation-related adverse events of propofol to those of midazolam in RCTs of cirrhotic patients undergoing UGIE. Based on (2.1.1) incidence of hypotension, (2.1.2) incidence of hypoxemia, or (2.1.3) incidence of bradycardia.

**(i) Hypotension.** Four of the selected RCTs [23–26] evaluated hypotension based on a systolic pressure below 90 mm Hg or a drop in blood pressure > 20% from baseline. In these studies, 19 of the 190 patients in the propofol groups and 13 of the 182 patients in the midazolam groups exhibited hypotension. The meta-analysis revealed no significant difference in the incidence of hypotension between the 2 groups (RR: 1.37; 95% CI: 0.69 to 2.73; *p* = 0.36; Fig. 4).

**(ii) Hypoxemia.** Hypoxemia was defined as saturation < 85% in two of the selected studies [1,25] and as < 90% in the three remaining studies [23,24,26]. The incidence of hypoxemia was 4.52% (10/221) in the propofol group and 2.83% (6/212) in the midazolam group. The meta-analysis revealed no significant difference between the two groups (RR: 1.30; 95% CI: 0.48 to 3.54; *p* = 0.61; Fig. 4).

**(iii) Bradycardia.** Two of the selected RCTs [24,25] evaluated the incidence of bradycardia [24–26] based on a heart rate (HR) < 50 beats per minute (bpm). A third RCT [26] defined bradycardia as an HR < 55 bpm or an HR < 75% of the baseline HR. The incidence of hypoxemia was 6% (9/150) in the propofol group and 2.86% (4/140) in the midazolam group. The meta-analysis revealed no difference between the two groups (RR: 1.64; 95% CI: 0.12 to 22.37; *p* = 0.71). Significant heterogeneity existed among the selected RCTs (*I* ^*2*^ = 76%; *p* = 0.04; Fig. 4).

**(iv) Deterioration of hepatic encephalopathy.** Three of the selected RCTs [1,23,24] evaluated the deterioration of minimal encephalopathy by using psychometric tests. Although a meta-analysis of these three studies was not possible because the evaluation methods used differed, all three of the studies recommended that propofol be used as an alternative to midazolam for sedating cirrhotic patients. Agrawal et al. [23] concluded that propofol sedation for UGIE caused no deterioration in psychometric scores calculated using the number connection tests (NCTs) A and B, the digit symbol test, the line tracing test, and the serial dotting test. However, significant deterioration was observed in the critical flicker frequency (CFF) at 30 and 60 min (*p* = 0.01) [23]. At the mean CFF time, midazolam caused significant deterioration of psychometric test scores and increased the CFF compared with propofol [23]. Khamaysi et al. [1] reported no overt hepatic encephalopathy after sedation, but acknowledged that sedation with midazolam might exacerbate subclinical encephalopathy, leaving patients more encephalopathic at the time of discharge than at admission. The cognitive function score did not differ significantly between the two groups. Propofol impacted minimal encephalopathy less than midazolam did (NCT: 87.5 ± 62 s to 74.2 ± 58 s for propofol vs 72.8 ± 62 s to 85.6 ± 72 s for midazolam; *p* < 0.01). The median discharge NCT scores for midazolam were severe, whereas the median discharge NCT scores for propofol were moderate [1]. Riphaus et al. [24] used the NCT and the portosystemic encephalopathy (PSE) test to evaluate minimal hepatic encephalopathy, and observed that propofol sedation did not cause acute deterioration of minimal hepatic encephalopathy. The median time to complete the NCT improved by 13% in the propofol group, but remained unchanged in the midazolam group. Similar results were observed regarding the PSE scores. The PSE score generally declined after sedation with midazolam, whereas the propofol group exhibited a significant improvement in the PSE score, possibly as the result of a learning effect [24].

### Sensitivity analysis

A sensitivity analysis including trials with low methodological quality [1,24] (i.e., inadequate description of the randomization and allocation concealment), or with a major research bias in terms of rescue medication [25] showed no significant difference in the anesthetic performance and the incidence of complications.

## Discussion

We systematically evaluated and compared the efficacy and safety of using propofol and midazolam to sedate cirrhotic patients undergoing UGIE. Our results indicated that propofol does not cause acute deterioration of minimal hepatic encephalopathy. Propofol provided shorter times to sedation and recovery than midazolam, and no increase in adverse events was observed.

A previous meta-analysis [17] revealed that propofol sedation during GIE was associated with a lower risk of cardiopulmonary adverse events compared with midazolam, meperidine, and/or fentanyl in the general population. Another meta-analysis showed that propofol was safe and effective for GIE and was associated with shorter recovery and discharge periods, higher post-anesthesia recovery scores, better sedation and greater patient cooperation than traditional sedation, without an increase in cardiopulmonary complications [18]. However, the impact of liver cirrhosis on the effects of sedatives in cirrhotic patients remains unclear. A pilot study evaluated the safety and efficacy of propofol use during endoscopy in Korean patients with cirrhosis, and determined that sedation with propofol was well tolerated in cirrhotic patients; specifically, no deterioration in psychomotor function, even in cirrhotic patients with hepatic encephalopathy, was observed [28]. Our study is the first meta-analysis to evaluate anesthesia for UGIE in cirrhotic patients. Our results indicated that propofol exhibits more favorable anesthetic performance than midazolam does in reducing performance and does not increase the risk of adverse events or deterioration of encephalopathy.

Previous studies have reported that the pharmacokinetics and protein binding of propofol are not markedly affected by moderate or uncomplicated cirrhosis [7,29,30]. Although recovery times were significantly longer and the volume of distribution at a steady state was significantly greater in the patients with cirrhosis than in those without cirrhosis, no significant difference in protein binding, total body clearance, or terminal elimination half-life was observed [31,32]. In the five RCTs selected for our study, 0.25 to 1 mg/kg of propofol was administered, and additional doses of 10 to 30 mg were administered as required. Such doses are similar to those used to sedate healthy patients undergoing GIE. Khamaysi et al. [1] and Riphaus et al. [24] reported that the anesthetic performance and the median PSE score change in cirrhotic patients receiving propofol were similar to noncirrhotic patients, and were superior to those receiving midazolam.

Cardiopulmonary adverse events, such as hypoxia, hypotension, arrhythmia, and apnea, are concerns regarding propofol sedation in cirrhotic patients because propofol exhibits greater suppressive cardiopulmonary effects than midazolam does. A previous meta-analysis compared using propofol combined with other sedative agents to sedate patients undergoing GIE with sedation using propofol alone, and determined that administering a higher dose of propofol did not increase the risk of cardiopulmonary adverse events [33]; this observation is consistent with our findings that propofol sedation does not increase the risk of bradycardia, hypotension, or hypoxemia compared with midazolam sedation. Sedatives used for UGIE can be administered by anesthesiologists, gastroenterologists, and trained registered nurses. Among the RCTs analyzed in the current study, sedatives were induced by anesthesiologists, gastroenterologists, and trained registered nurses in two [1,23], one [26], and one [25] of the trials, respectively, and our meta-analysis revealed no significant difference in the incidence of major adverse events between these studies.

Our meta-analysis revealed that the procedure times in the midazolam groups were shorter than those in the propofol group, whereas propofol provided shorter times to sedation and recovery. The times to sedation and recovery are direct indicators of anesthetic performance, whereas procedure time is influenced by additional factors such as endoscopic findings, the need for varix ligation, and the experience of the endoscopist. Riphaus et al. observed that band ligation was more frequent in the propofol group than in the midazolam group [17]. Agrawal et al. reported that the number of patients with large varices in the propofol group (*n* = 18) was higher than that in midazolam group (*n* = 14) [16]. Future studies comparing UGIE procedure time in patients sedated with propofol or midazolam are warranted to clarify these findings.

The severity of liver cirrhosis is an independent variable in determining duration of drug action. In our included studies, one mainly enrolled patients with CP classification A [1], one recruited patients with CP classification B [23], and the other three trials included both CP classification A and B patients [24–26]. However, regardless of how variable the severity of cirrhosis was, propofol provided faster time to sedation and time to recovery than those of midazolam. Because of the limited numbers of cirrhotic C patients recruited, the outcomes of propofol and midazolam in these patients cannot be concluded and needs further investigations.

Various factors might have contributed to the high level of heterogeneity observed among the RCTs analyzed in our study. First, the CP scores varied considerably among the five studies. Second, the anesthetic interventions were not identical across all of the studies. Therefore, clinical factors, such as opioid dosages and the experience level of the anesthesiologists, might have contributed to heterogeneity. Third, differences in the definitions used for the various outcomes and adverse events might also have contributed to heterogeneity.

Our current findings are also subject to certain limitations. Variation in population characteristics, low patient numbers, unbalanced patient numbers among groups, differences in anesthetic agents and dosages, variation in outcome analyses, and the methodological weaknesses inherent in the selected RCTs might limit inferences based on our results. Furthermore, in one of the RCTs, more esophageal varices occurred in the propofol group than in the midazolam group and more portal gastropathy occurred in the midazolam group than in the propofol group [1]; these results might have confounded the outcome analysis. Moreover, most of the RCTs included in our current study examined patients with mild to moderate cirrhosis. Therefore, our findings might not be applicable to patients with severe cirrhosis.

In conclusion, our meta-analysis revealed that, compared with midazolam sedation, cirrhotic patients undergoing UGIE who are sedated using propofol exhibit more rapid sedation, faster recovery, earlier discharge, and more favorable psychometric and cognitive scores than do those sedated using midazolam, with no increased risk of hypotension, bradycardia, or hypoxemia. Therefore, we recommend using propofol as an alternative to midazolam in sedating cirrhotic patients undergoing UGIE.

## Supporting Information

S1 PRISMA Checklist (DOCX)

S1 Excluded Studies List (DOCX)
