# Supplementary material for: Propofol versus Midazolam for Upper Gastrointestinal Endoscopy in Cirrhotic Patients: A Meta-Analysis of Randomized Controlled Trials
Source: PLoS One. 2015 Feb 3;10(2):e0117585. doi: 10.1371/journal.pone.0117585 (PMC4315567; doi:10.1371/journal.pone.0117585)
Supplement: S1 Excluded Studies List — (DOCX) [file pone.0117585.s002.docx]

**Supporting information—the 43 excluded studies**

1. Different comparison: [[1-28](#_ENREF_1)]
2. Not randomized:

Prospective: [[29](#_ENREF_29)]

Retrospective cohort: [[30-32](#_ENREF_30)]

Meta-analysis: [[33](#_ENREF_33), [34](#_ENREF_34)]

Review: [[35-43](#_ENREF_35)]

1. Kilgert, B., et al., *Prospective long-term assessment of sedation-related adverse events and patient satisfaction for upper endoscopy and colonoscopy.* Digestion, 2014. **90**(1): p. 42-8.

2. Eberl, S., et al., *Is "really conscious" sedation with solely an opioid an alternative to every day used sedation regimes for colonoscopies in a teaching hospital? Midazolam/fentanyl, propofol/alfentanil, or alfentanil only for colonoscopy: a randomized trial.* Tech Coloproctol, 2014. **18**(8): p. 745-52.

3. Watkins, T.J., et al., *Evaluation of postprocedure cognitive function using 3 distinct standard sedation regimens for endoscopic procedures.* AANA J, 2014. **82**(2): p. 133-9.

4. Oh, J.E., H.J. Lee, and Y.H. Lee, *Propofol versus Midazolam for Sedation during Esophagogastroduodenoscopy in Children.* Clin Endosc, 2013. **46**(4): p. 368-72.

5. Lera dos Santos, M.E., et al., *Deep sedation during gastrointestinal endoscopy: propofol-fentanyl and midazolam-fentanyl regimens.* World J Gastroenterol, 2013. **19**(22): p. 3439-46.

6. Zuo, X.L., et al., *Propofol vs midazolam plus fentanyl for upper gastrointestinal endomicroscopy: a randomized trial.* World J Gastroenterol, 2012. **18**(15): p. 1814-21.

7. Levitzky, B.E., et al., *Moderate sedation for elective upper endoscopy with balanced propofol versus fentanyl and midazolam alone: a randomized clinical trial.* Endoscopy, 2012. **44**(1): p. 13-20.

8. Kiriyama, S., et al., *Safe and effective sedation in endoscopic submucosal dissection for early gastric cancer: a randomized comparison between propofol continuous infusion and intermittent midazolam injection.* J Gastroenterol, 2010. **45**(8): p. 831-7.

9. Schilling, D., et al., *Sedation with propofol for interventional endoscopy by trained nurses in high-risk octogenarians: a prospective, randomized, controlled study.* Endoscopy, 2009. **41**(4): p. 295-8.

10. Dewitt, J., et al., *Nurse-administered propofol sedation compared with midazolam and meperidine for EUS: a prospective, randomized trial.* Gastrointest Endosc, 2008. **68**(3): p. 499-509.

11. Kongkam, P., et al., *Propofol infusion versus intermittent meperidine and midazolam injection for conscious sedation in ERCP.* J Gastrointestin Liver Dis, 2008. **17**(3): p. 291-7.

12. Mandel, J.E., et al., *A randomized, controlled, double-blind trial of patient-controlled sedation with propofol/remifentanil versus midazolam/fentanyl for colonoscopy.* Anesth Analg, 2008. **106**(2): p. 434-9, table of contents.

13. Agostoni, M., et al., *Midazolam and pethidine versus propofol and fentanyl patient controlled sedation/analgesia for upper gastrointestinal tract ultrasound endoscopy: a prospective randomized controlled trial.* Dig Liver Dis, 2007. **39**(11): p. 1024-9.

14. Meining, A., et al., *The effect of sedation on the quality of upper gastrointestinal endoscopy: an investigator-blinded, randomized study comparing propofol with midazolam.* Endoscopy, 2007. **39**(4): p. 345-9.

15. Riphaus, A., et al., *Quality of psychomotor recovery after propofol sedation for routine endoscopy: a randomized and controlled study.* Endoscopy, 2006. **38**(7): p. 677-83.

16. Riphaus, A., N. Stergiou, and T. Wehrmann, *Sedation with propofol for routine ERCP in high-risk octogenarians: a randomized, controlled study.* Am J Gastroenterol, 2005. **100**(9): p. 1957-63.

17. Ulmer, B.J., et al., *Propofol versus midazolam/fentanyl for outpatient colonoscopy: administration by nurses supervised by endoscopists.* Clin Gastroenterol Hepatol, 2003. **1**(6): p. 425-32.

18. Bright, E., et al., *Patient-controlled sedation for colonoscopy: a randomized trial comparing patient-controlled administration of propofol and alfentanil with physician-administered midazolam and pethidine.* Endoscopy, 2003. **35**(8): p. 683-7.

19. Khoshoo, V., et al., *Propofol versus midazolam plus meperidine for sedation during ambulatory esophagogastroduodenoscopy.* J Pediatr Gastroenterol Nutr, 2003. **37**(2): p. 146-9.

20. Vargo, J.J., et al., *Gastroenterologist-administered propofol versus meperidine and midazolam for advanced upper endoscopy: a prospective, randomized trial.* Gastroenterology, 2002. **123**(1): p. 8-16.

21. Sipe, B.W., et al., *Propofol versus midazolam/meperidine for outpatient colonoscopy: administration by nurses supervised by endoscopists.* Gastrointest Endosc, 2002. **55**(7): p. 815-25.

22. Koshy, G., et al., *Propofol versus midazolam and meperidine for conscious sedation in GI endoscopy.* Am J Gastroenterol, 2000. **95**(6): p. 1476-9.

23. Jung, M., et al., *Improved sedation in diagnostic and therapeutic ERCP: propofol is an alternative to midazolam.* Endoscopy, 2000. **32**(3): p. 233-8.

24. Hofmann, C., et al., *[Propofol for sedation in gastroscopy--a randomized comparison with midazolam].* Z Gastroenterol, 1999. **37**(7): p. 589-95.

25. Wehrmann, T., et al., *Efficacy and safety of intravenous propofol sedation during routine ERCP: a prospective, controlled study.* Gastrointest Endosc, 1999. **49**(6): p. 677-83.

26. Carlsson, U. and P. Grattidge, *Sedation for upper gastrointestinal endoscopy: a comparative study of propofol and midazolam.* Endoscopy, 1995. **27**(3): p. 240-3.

27. Chin, N.M., H.Y. Tai, and M.K. Chin, *Intravenous sedation for upper gastrointestinal endoscopy: Midazolam versus propofol.* Singapore Med J, 1992. **33**(5): p. 478-80.

28. Patterson, K.W., et al., *Propofol sedation for outpatient upper gastrointestinal endoscopy: comparison with midazolam.* Br J Anaesth, 1991. **67**(1): p. 108-11.

29. Lightdale, J.R., et al., *Efficiency of propofol versus midazolam and fentanyl sedation at a pediatric teaching hospital: a prospective study.* Gastrointest Endosc, 2008. **67**(7): p. 1067-75.

30. Cohen, S., et al., *Propofol for pediatric colonoscopy: the experience of a large, tertiary care pediatric hospital.* Am J Ther, 2014. **21**(6): p. 509-11.

31. Lordan, J.T., et al., *A retrospective analysis of benzodiazepine sedation vs. propofol anaesthesia in 252 patients undergoing endoscopic retrograde cholangiopancreatography.* HPB (Oxford), 2011. **13**(3): p. 174-7.

32. Nayar, D.S., et al., *Comparison of propofol deep sedation versus moderate sedation during endosonography.* Dig Dis Sci, 2010. **55**(9): p. 2537-44.

33. McQuaid, K.R. and L. Laine, *A systematic review and meta-analysis of randomized, controlled trials of moderate sedation for routine endoscopic procedures.* Gastrointest Endosc, 2008. **67**(6): p. 910-23.

34. Qadeer, M.A., et al., *Propofol versus traditional sedative agents for gastrointestinal endoscopy: a meta-analysis.* Clin Gastroenterol Hepatol, 2005. **3**(11): p. 1049-56.

35. Kiriyama, S., H. Naitoh, and H. Kuwano, *Propofol sedation during endoscopic treatment for early gastric cancer compared to midazolam.* World J Gastroenterol, 2014. **20**(34): p. 11985-90.

36. Moon, S.H., *Sedation regimens for gastrointestinal endoscopy.* Clin Endosc, 2014. **47**(2): p. 135-40.

37. Manickam, P., Z. Kanaan, and K. Zakaria, *Conscious sedation: a dying practice?* World J Gastroenterol, 2013. **19**(28): p. 4633-4.

38. Poulos, J.E., P.T. Kalogerinis, and J.N. Caudle, *Propofol compared with combination propofol or midazolam/fentanyl for endoscopy in a community setting.* AANA J, 2013. **81**(1): p. 31-6.

39. Triantafillidis, J.K., et al., *Sedation in gastrointestinal endoscopy: current issues.* World J Gastroenterol, 2013. **19**(4): p. 463-81.

40. Garewal, D., et al., *Sedative techniques for endoscopic retrograde cholangiopancreatography.* Cochrane Database Syst Rev, 2012. **6**: p. CD007274.

41. van Beek, E.J. and P.L. Leroy, *Safe and effective procedural sedation for gastrointestinal endoscopy in children.* J Pediatr Gastroenterol Nutr, 2012. **54**(2): p. 171-85.

42. El Chafic, A.H., G. Eckert, and D.K. Rex, *Prospective description of coughing, hemodynamic changes, and oxygen desaturation during endoscopic sedation.* Dig Dis Sci, 2012. **57**(7): p. 1899-907.

43. Kapoor, H., *Anaesthesia for endoscopic retrograde cholangiopancreatography.* Acta Anaesthesiol Scand, 2011. **55**(8): p. 918-26.
